# Supplementary figures and images for: RAD51 restricts DNA over-replication from re-activated origins
Source: EMBO J. 2024 Feb 15;43(6):7. doi: 10.1038/s44318-024-00038-z (PMC10942984; doi:10.1038/s44318-024-00038-z)

Figure 1C

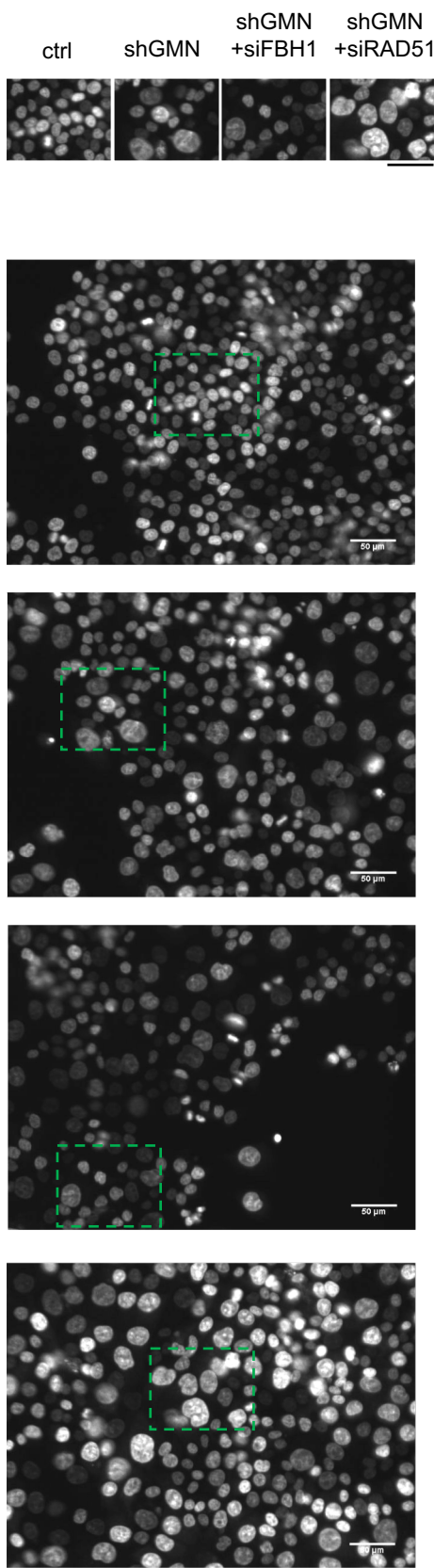

Supplement: Supplementary file 6 — Source Data Fig. 1 [file 44318_2024_38_MOESM6_ESM.zip › IF_Figure 1.pdf]

Fig 1B

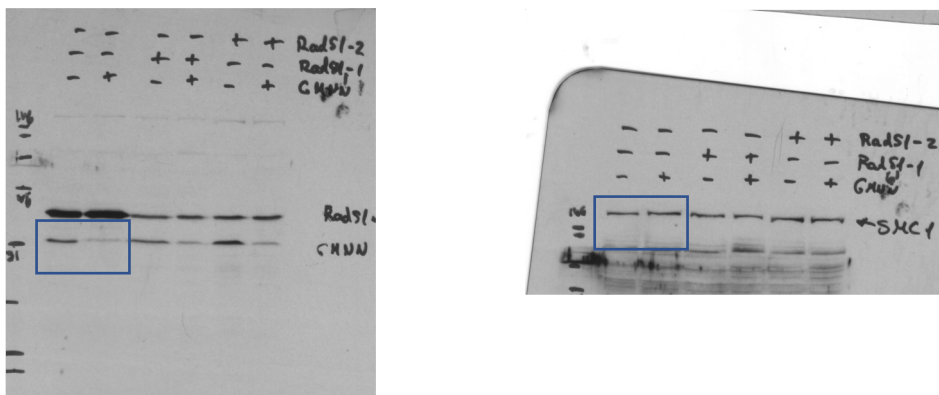

Fig 1D

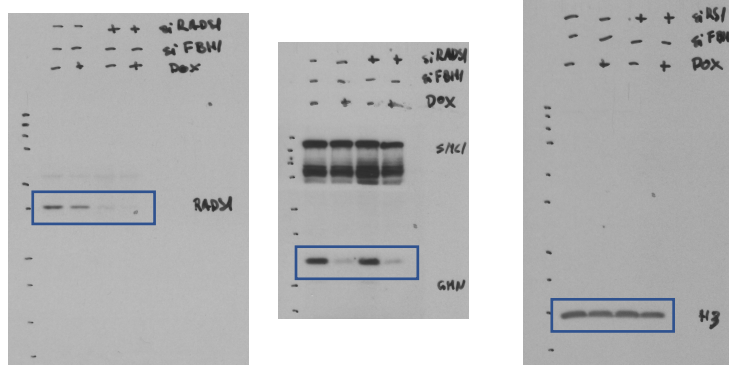

Supplement: Supplementary file 6 — Source Data Fig. 1 [file 44318_2024_38_MOESM6_ESM.zip › WB_Figure 1.pdf]

Figure 2A  
(left)

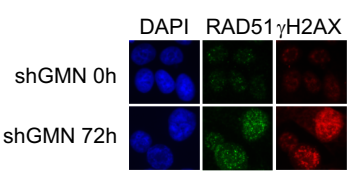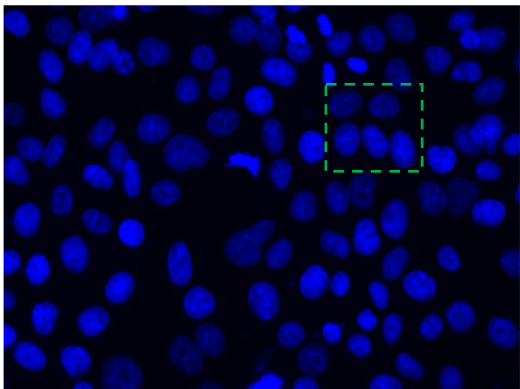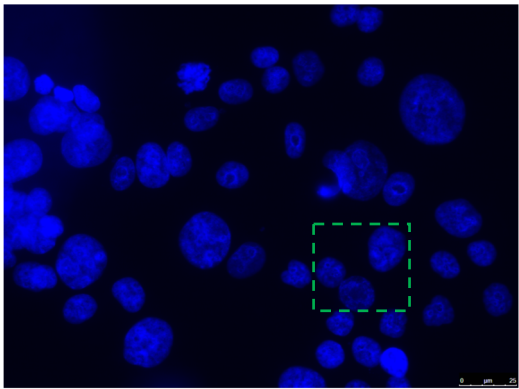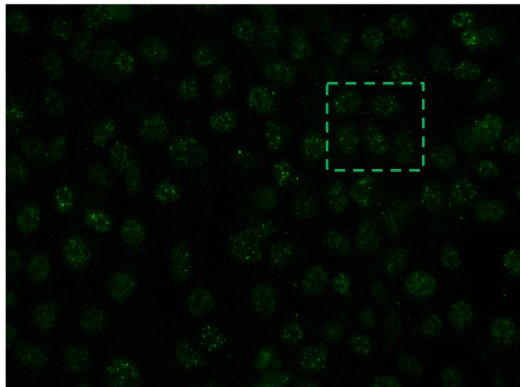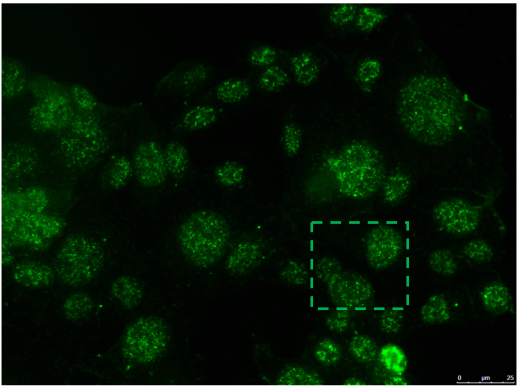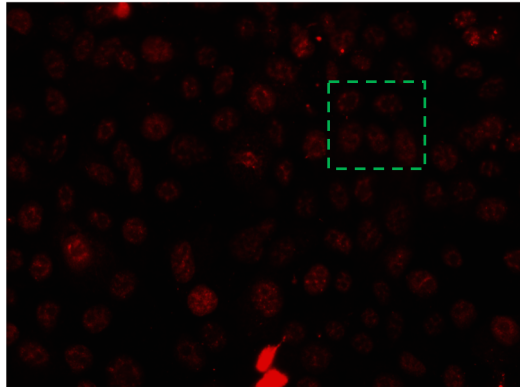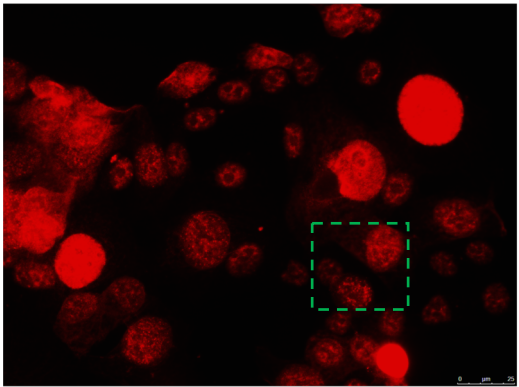

Figure 2A  
(right)

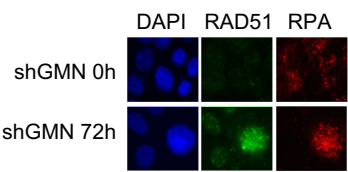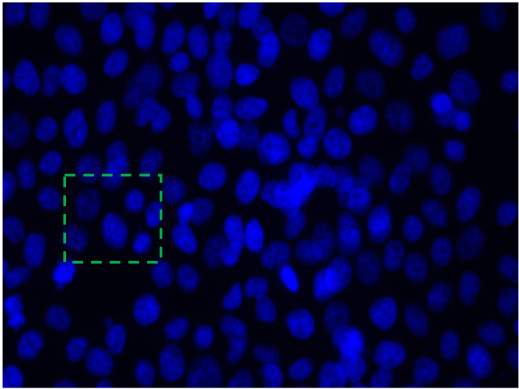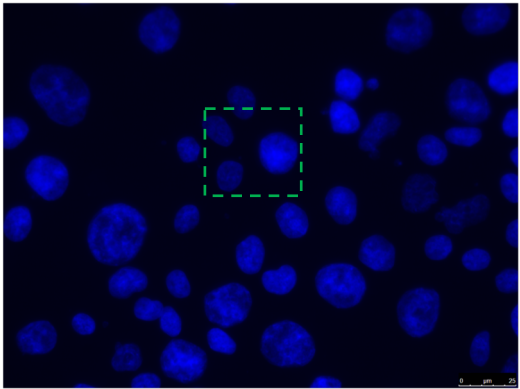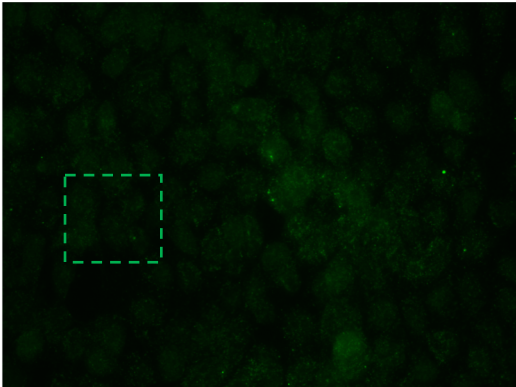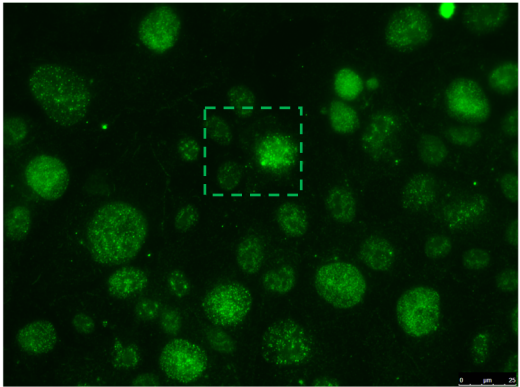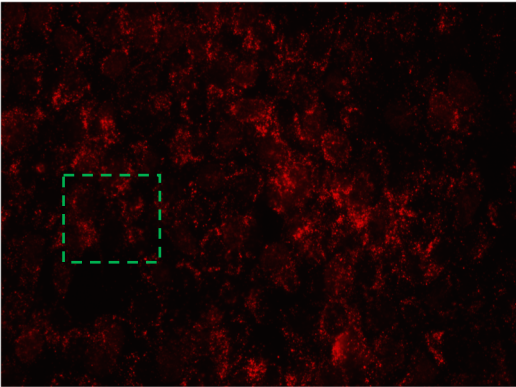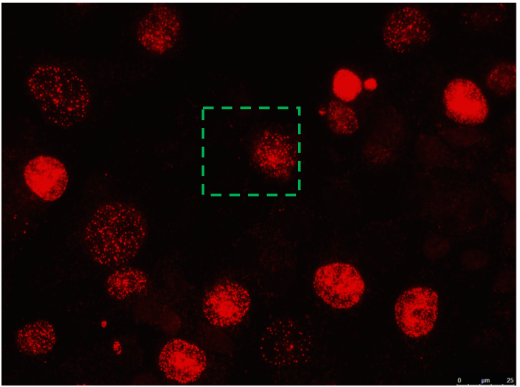

Supplement: Supplementary file 7 — Source Data Fig. 2 [file 44318_2024_38_MOESM7_ESM.zip › IF_Figure 2.pdf]

Figure 2C

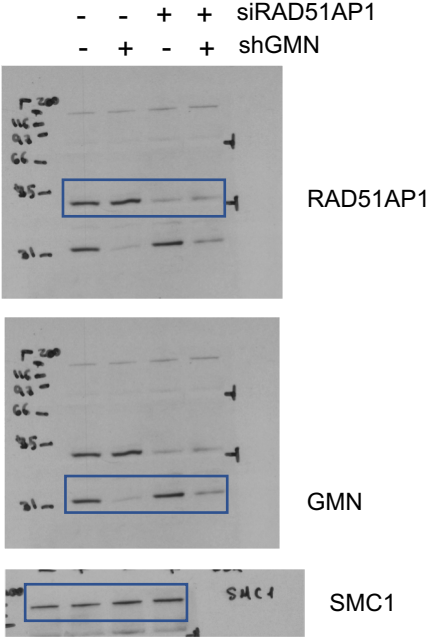

Figure 2D

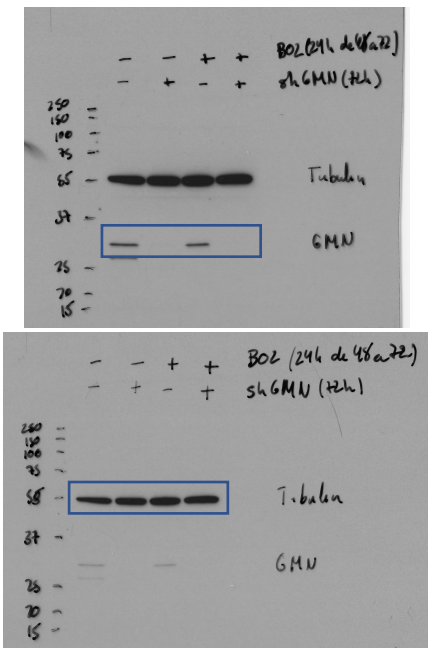

Figure 2E

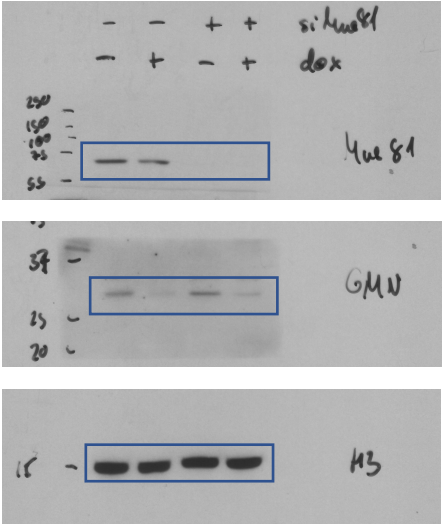

Supplement: Supplementary file 7 — Source Data Fig. 2 [file 44318_2024_38_MOESM7_ESM.zip › WB_Figure 2.pdf]

Figure 3A

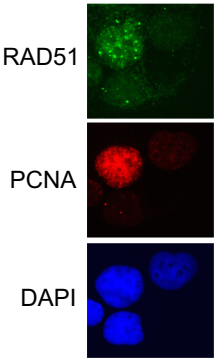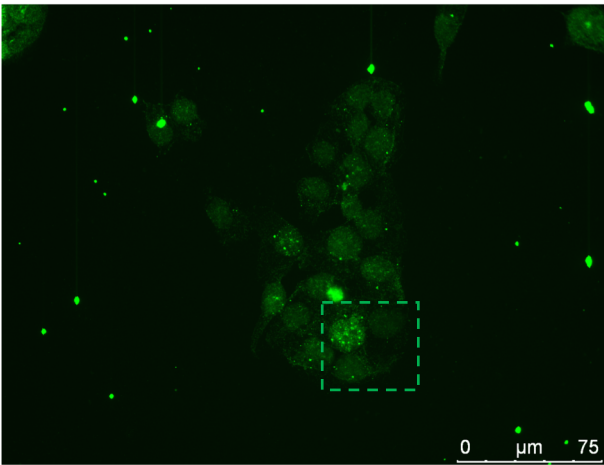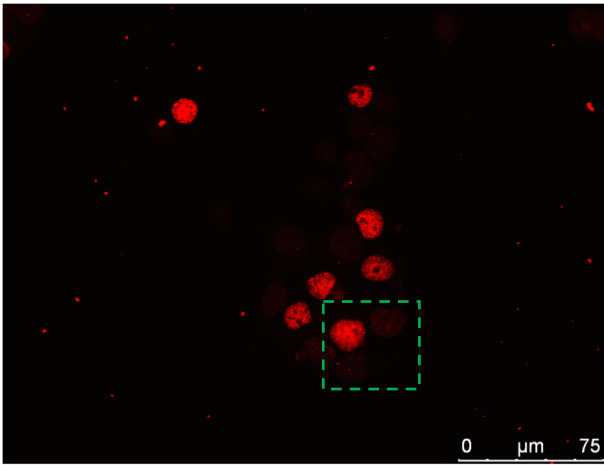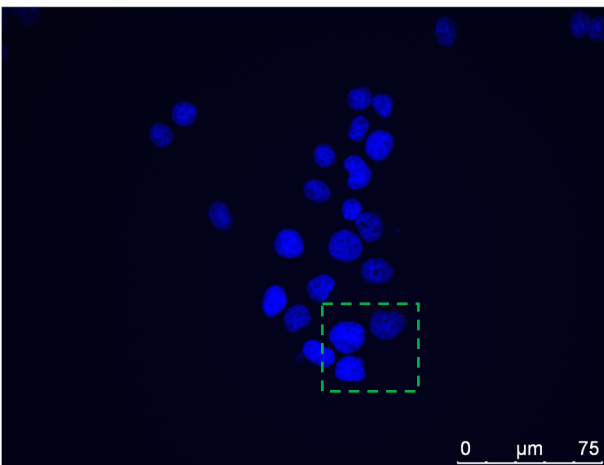

Figure 3C

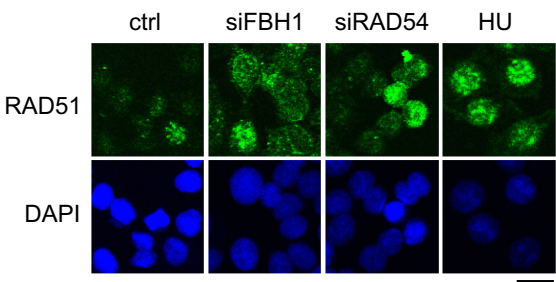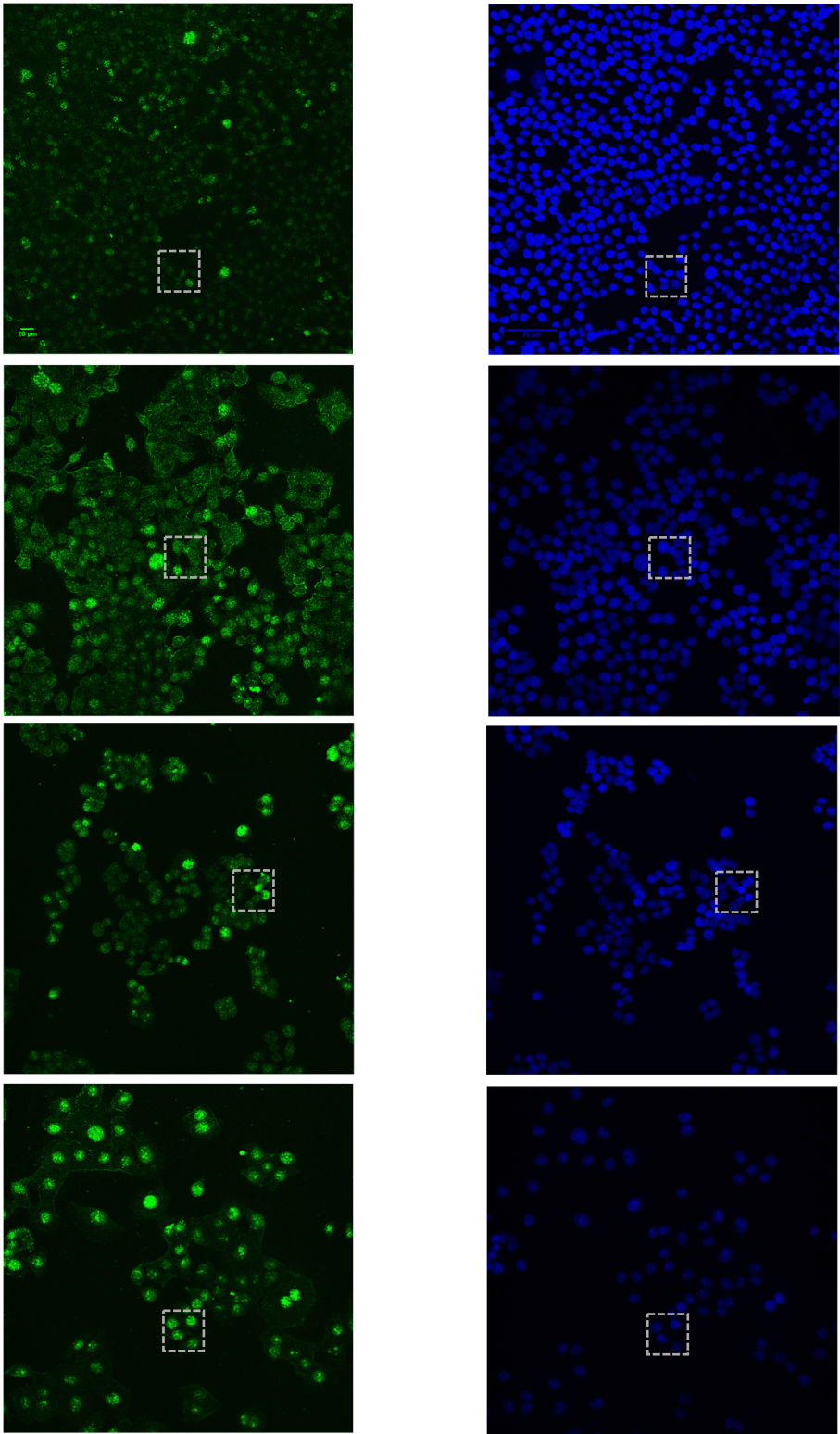

Supplement: Supplementary file 8 — Source Data Fig. 3 [file 44318_2024_38_MOESM8_ESM.zip › IF_Figure 3.pdf]

Figure 3B

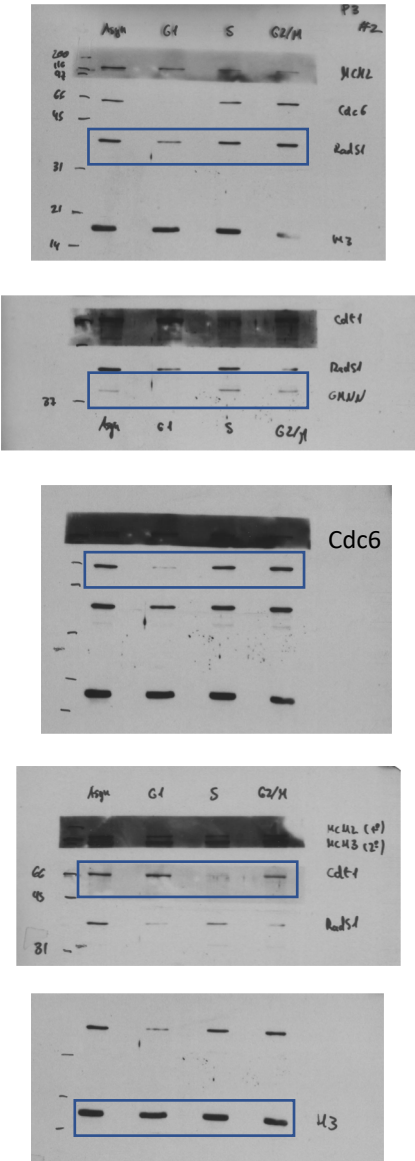

Figure 3D

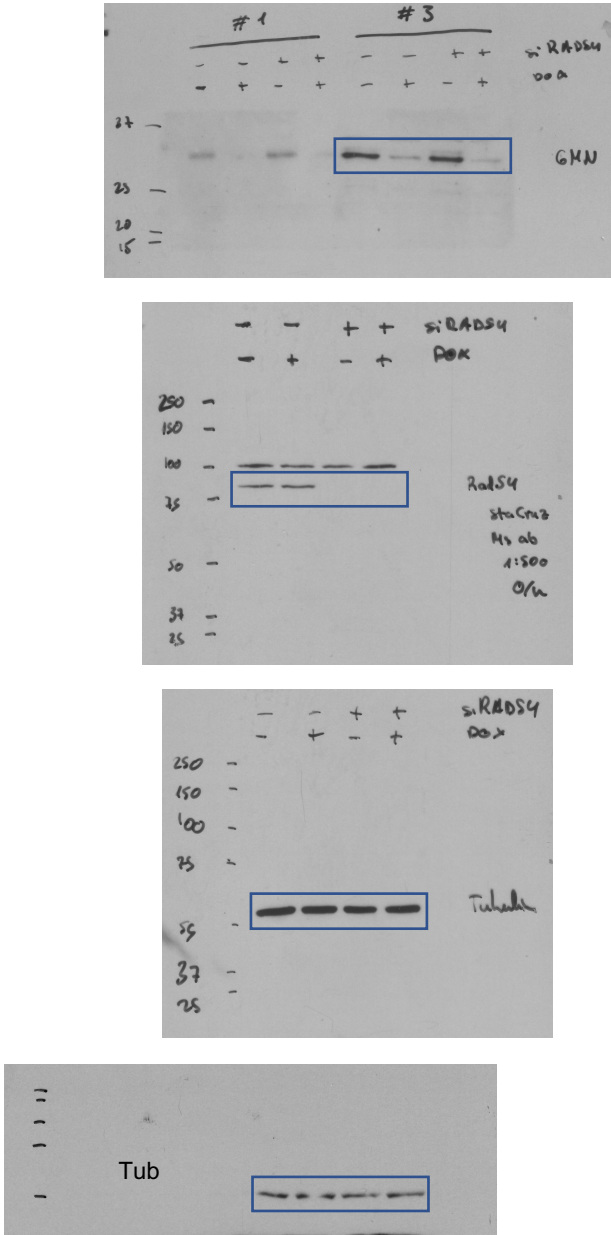

Supplement: Supplementary file 8 — Source Data Fig. 3 [file 44318_2024_38_MOESM8_ESM.zip › WB_Figure 3.pdf]

Figure 4A

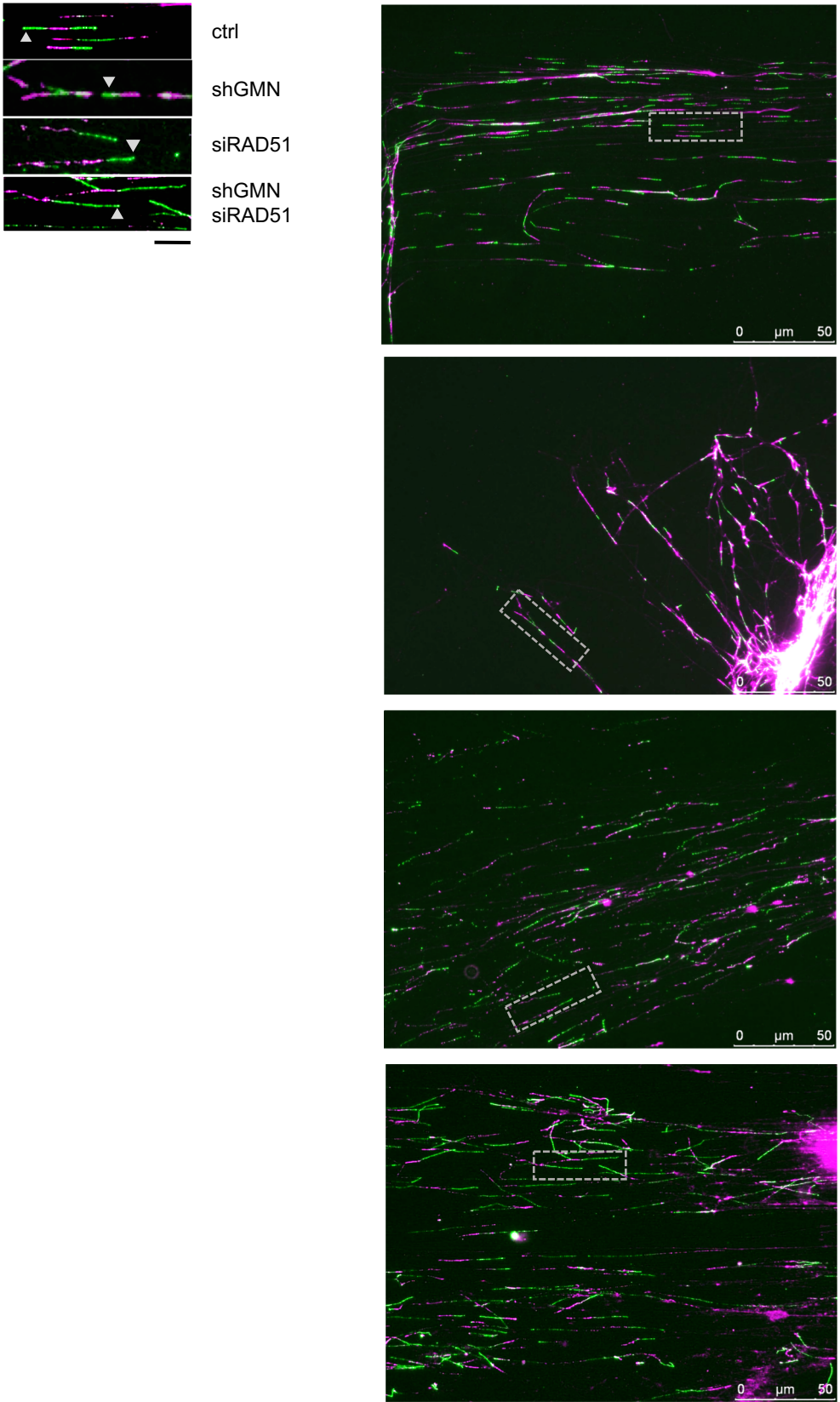

Figure 4E

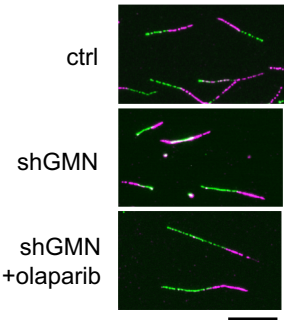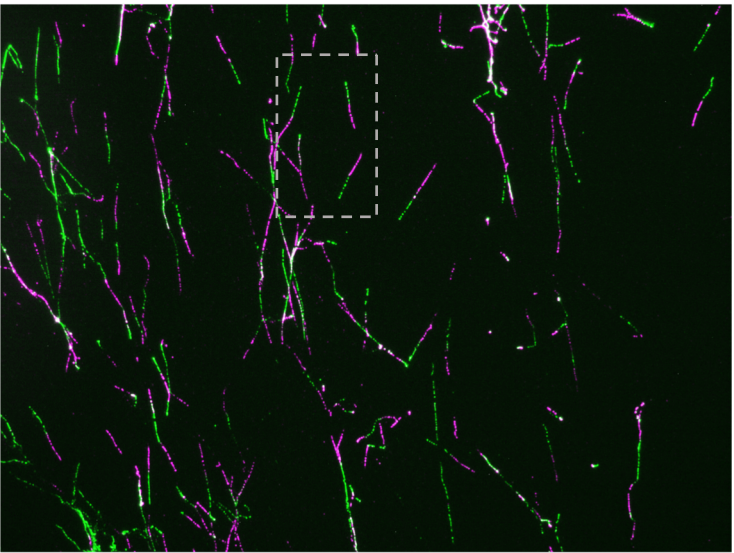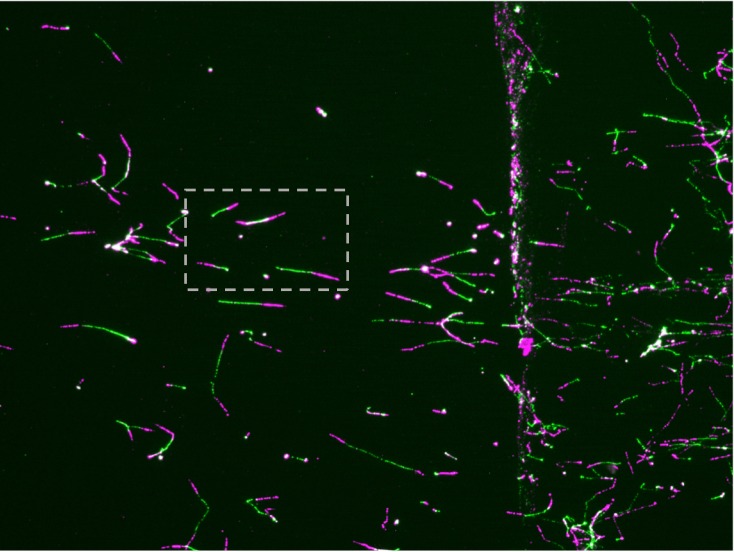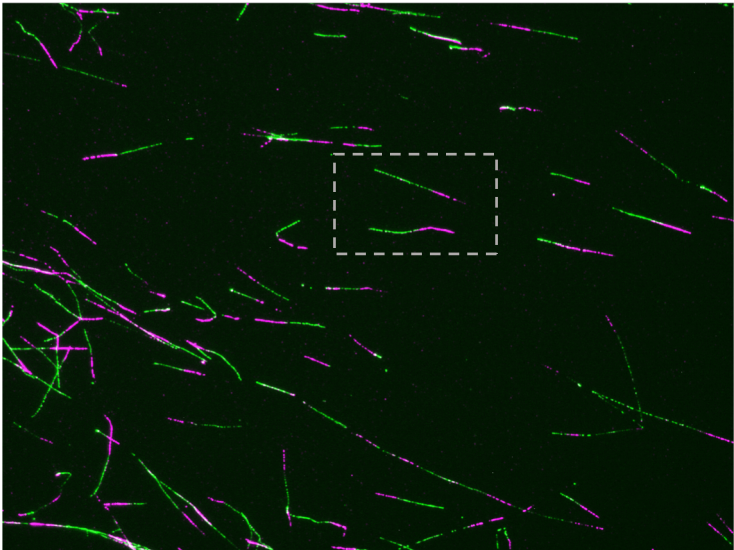

Figure 4G

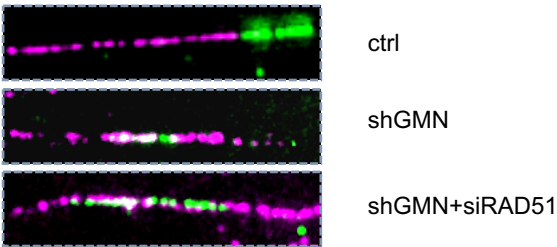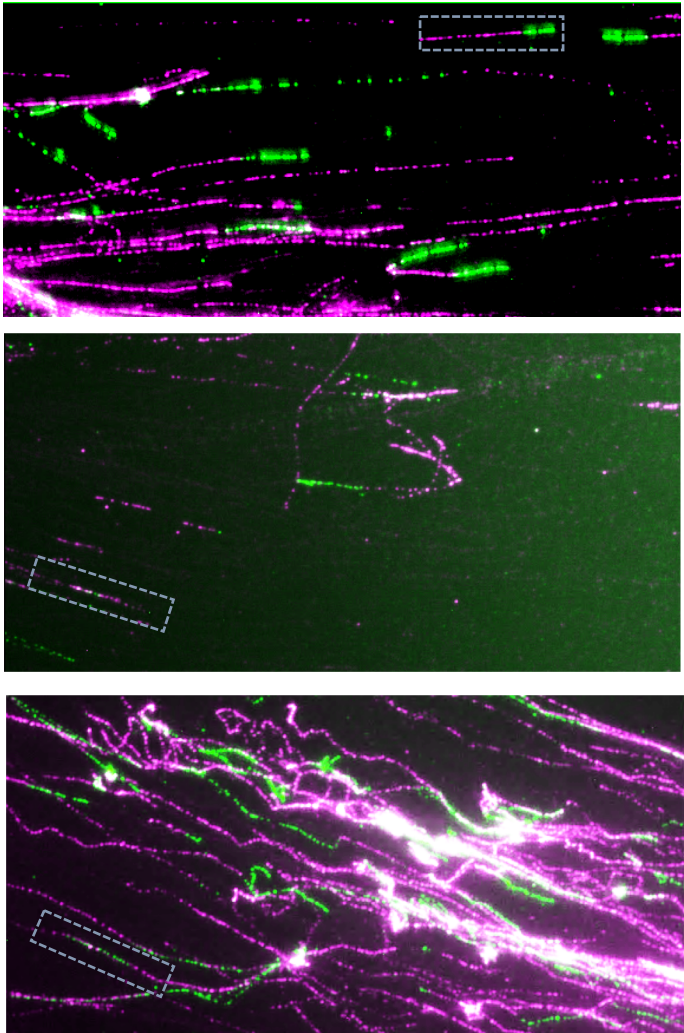

Supplement: Supplementary file 9 — Source Data Fig. 4 [file 44318_2024_38_MOESM9_ESM.zip › DNA Fibers_Figure 4.pdf]

Figure 5A

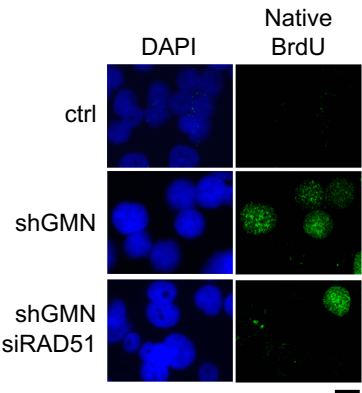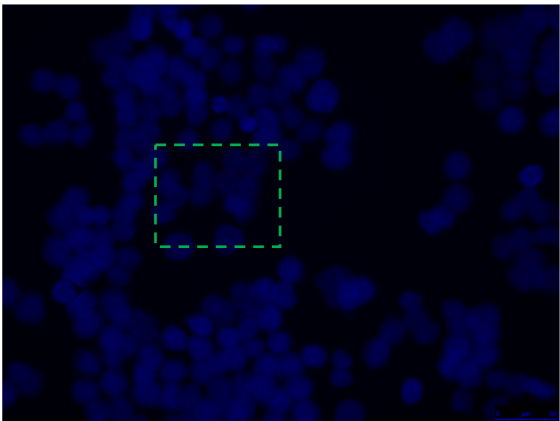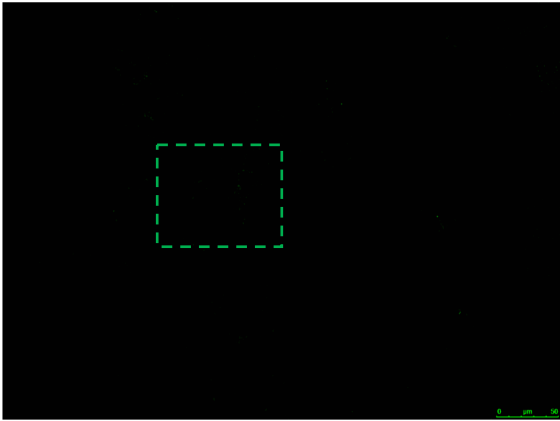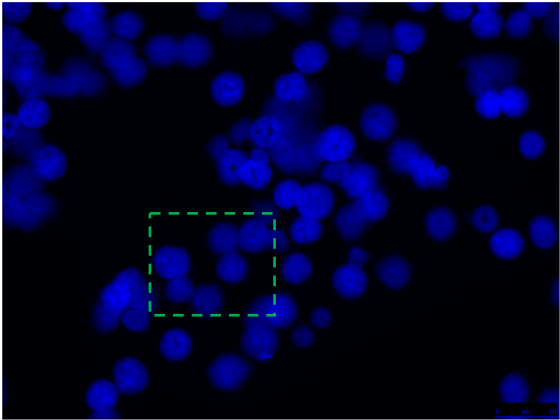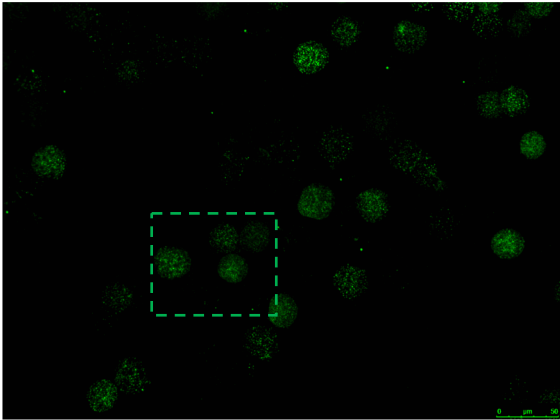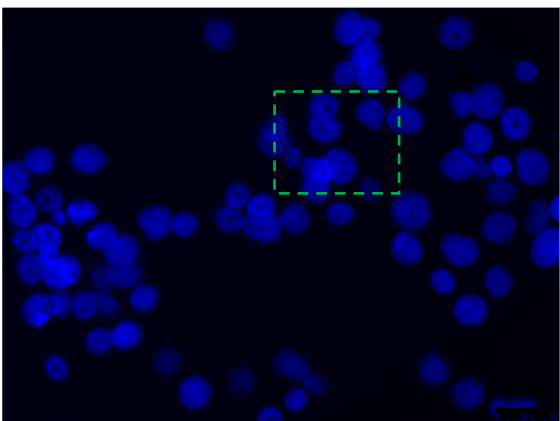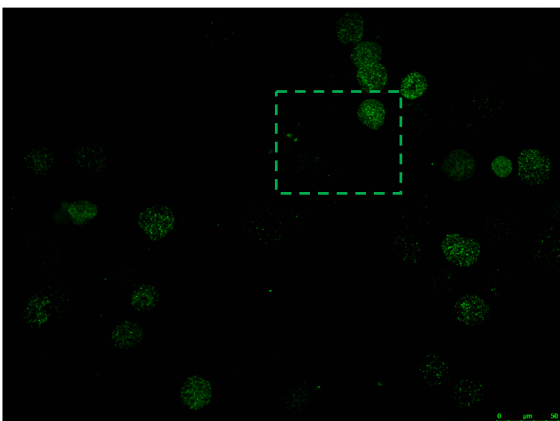

Figure 5B

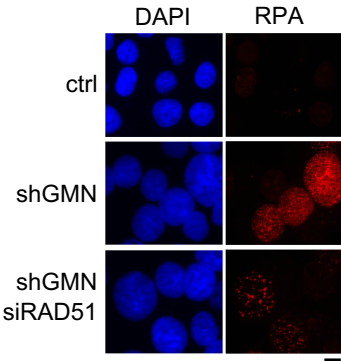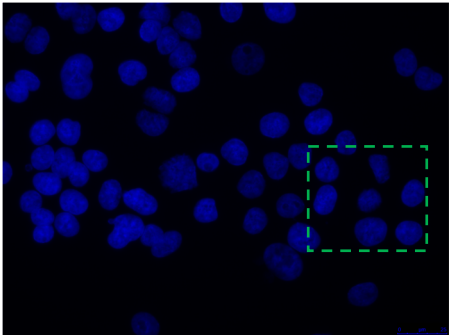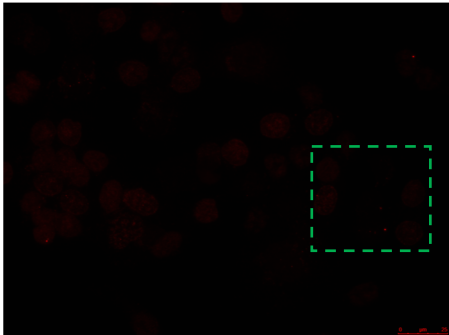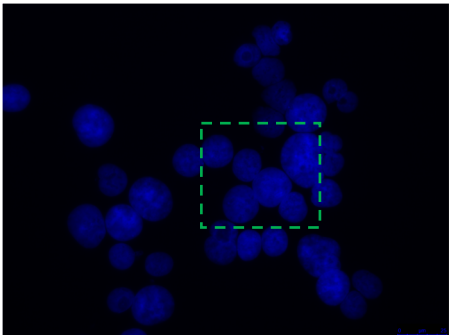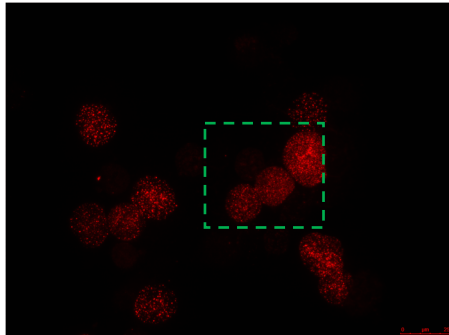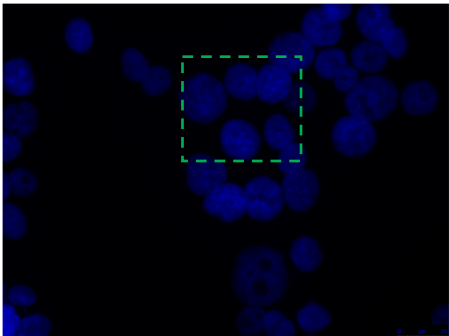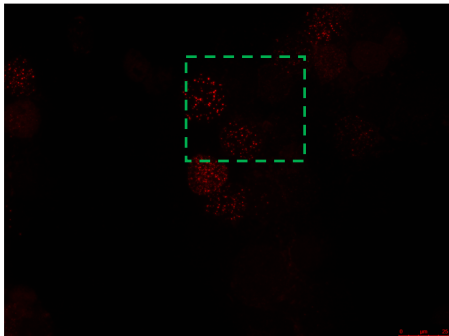

Supplement: Supplementary file 10 — Source Data Fig. 5 [file 44318_2024_38_MOESM10_ESM.zip › IF_Figure 5.pdf]

Figure 5C

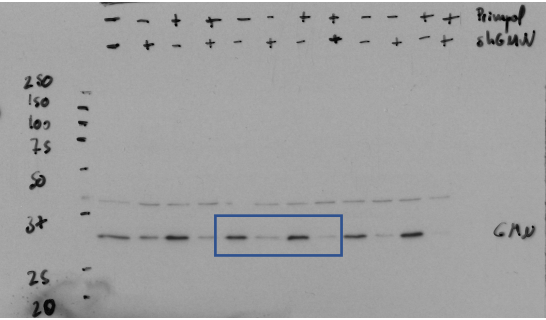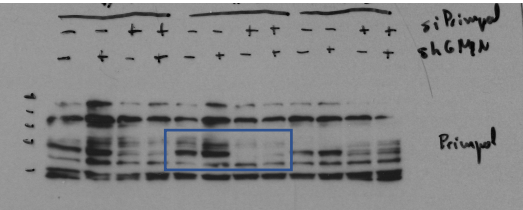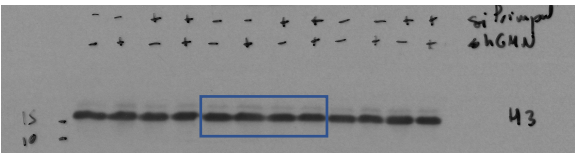

Figure 5D

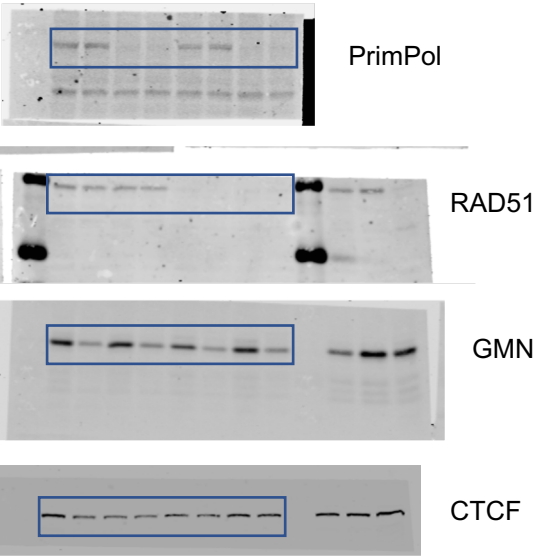

Figure 5E

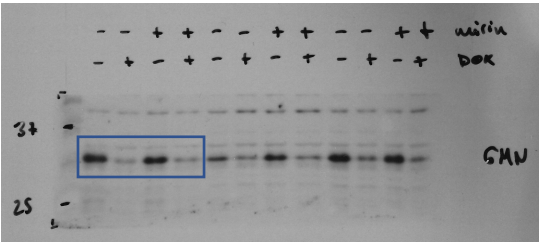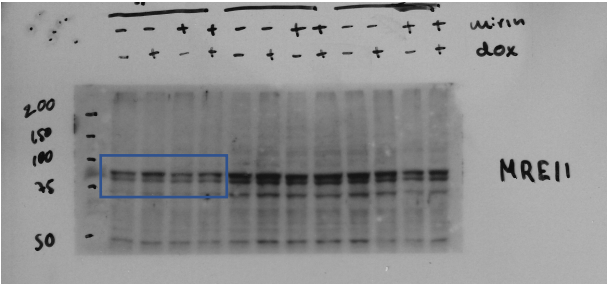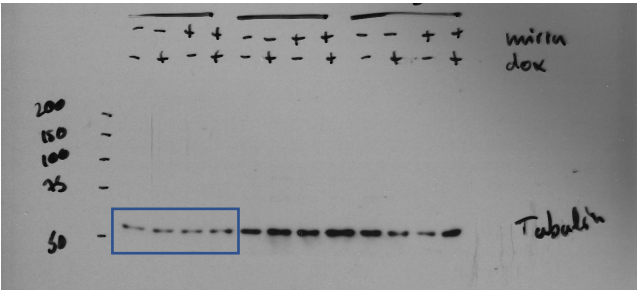

Supplement: Supplementary file 10 — Source Data Fig. 5 [file 44318_2024_38_MOESM10_ESM.zip › WB_Figure 5.pdf]
