# Supplementary material for: RAD51 restricts DNA over-replication from re-activated origins
Source: EMBO J. 2024 Feb 15;43(6):7. doi: 10.1038/s44318-024-00038-z (PMC10942984; doi:10.1038/s44318-024-00038-z)

Fig EV1B

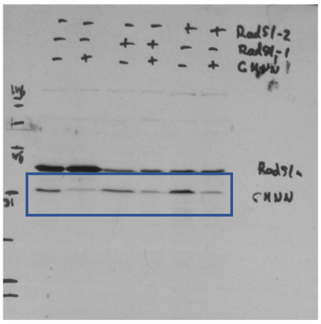

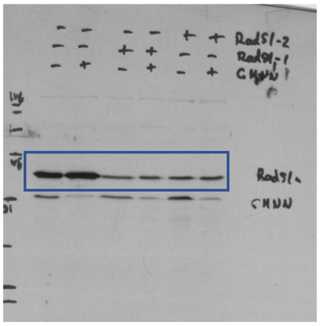

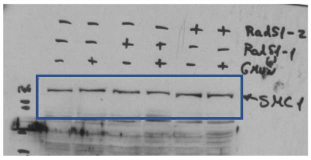

Fig EV1C

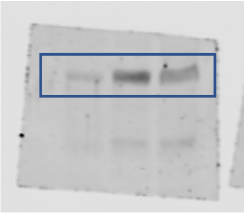

CDT1

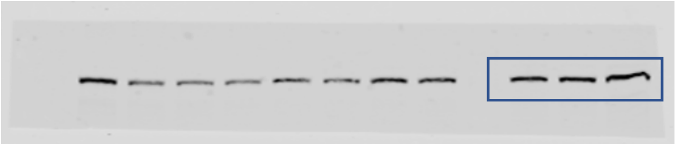

CTCF

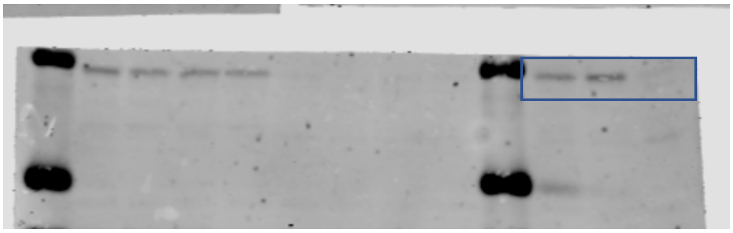

RAD51

Fig EV1D

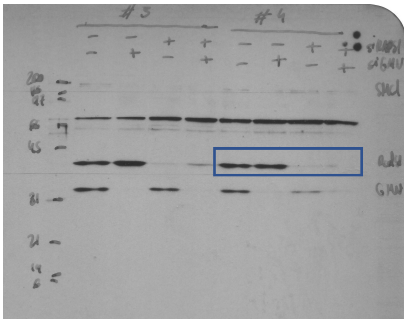

RAD51

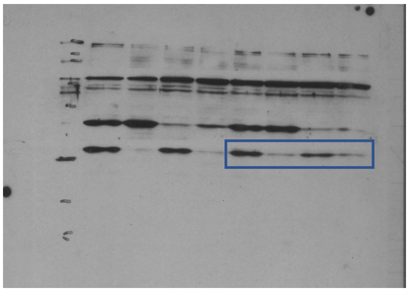

GMN

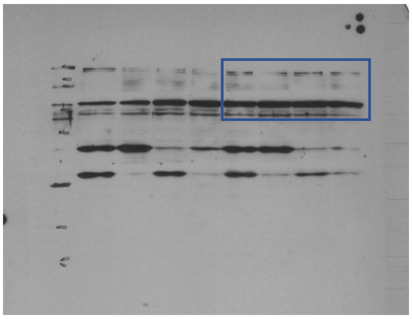

SMC1

Fig EV1E

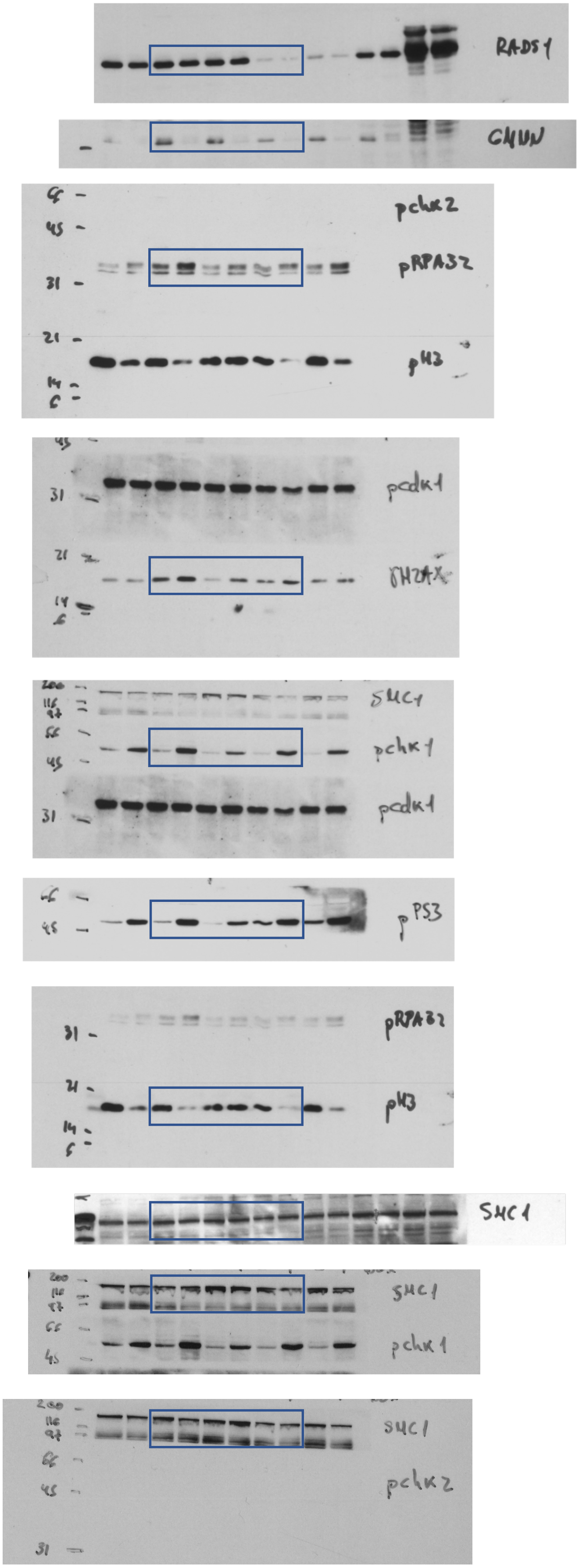
RAD51
pchk2
pRPA32
pH3
pcdk1
γH2AX
SMC1
pchk1
pcdk1
pP53
pRPA32
pH3
SMC1
SMC1
pchk1
SMC1
pchk2

Fig EV2A

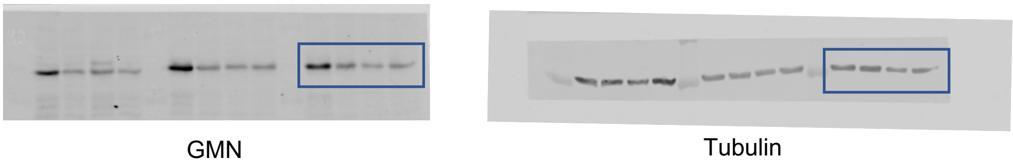

Fig EV2B

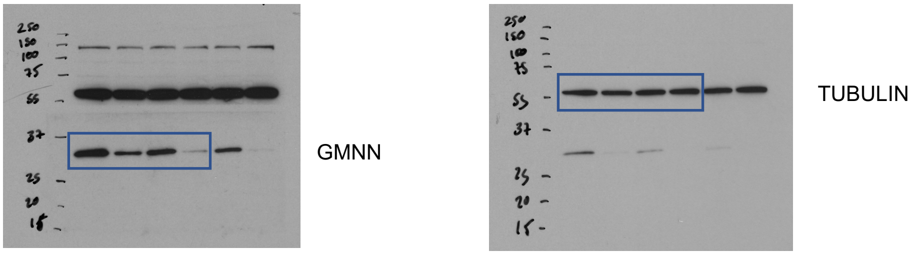

Fig EV2E

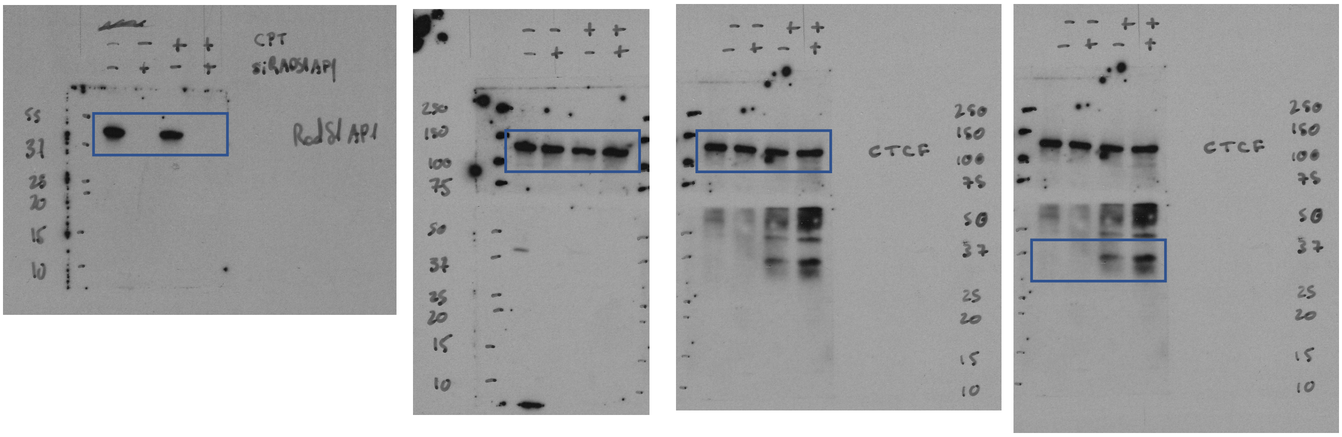

Fig EV2F

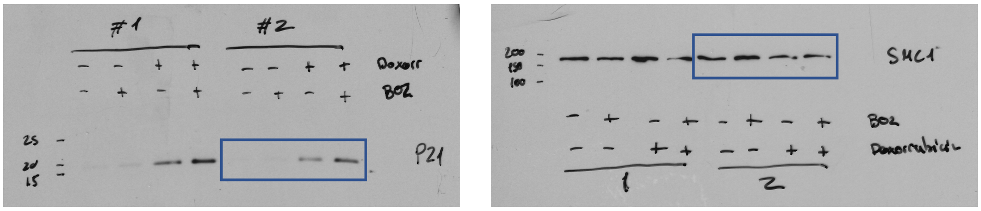

Fig EV4D

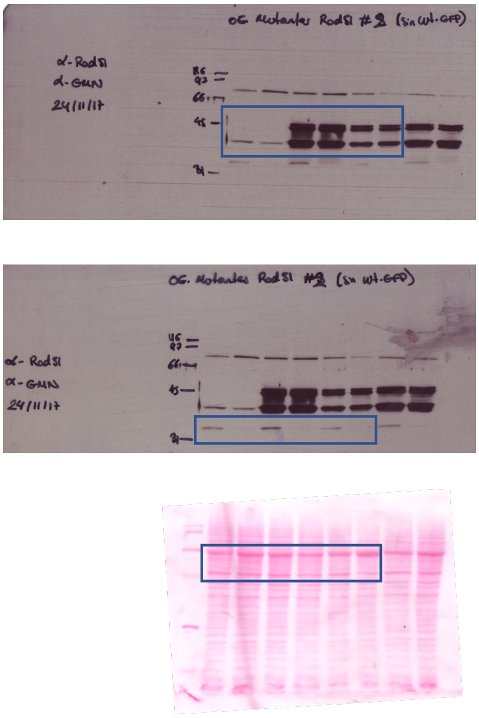

Fig EV5B

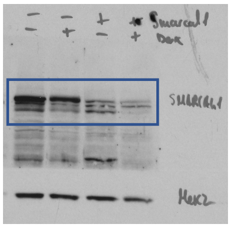

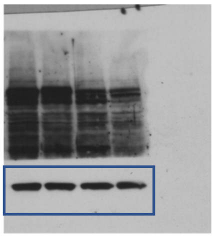

MEK2

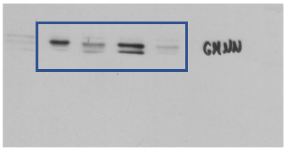

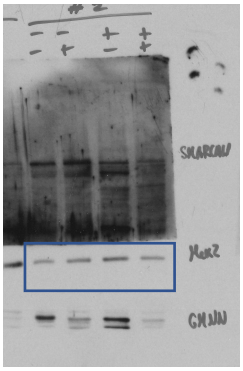

Fig EV5C

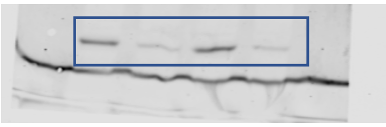

GMN

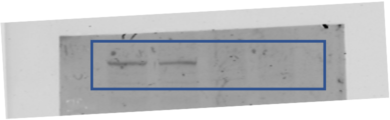

HLTF

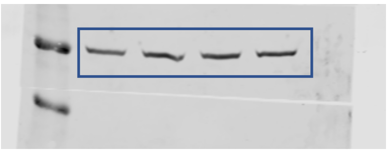

Tubulin

Fig EV6D

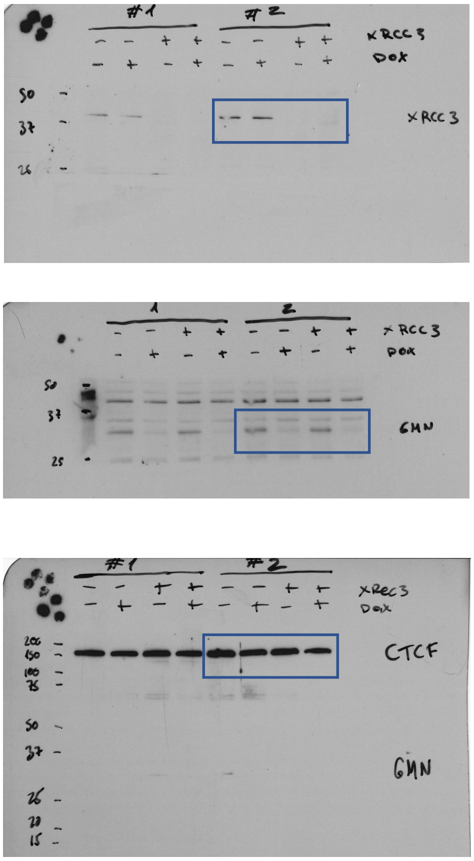

Fig EV6E

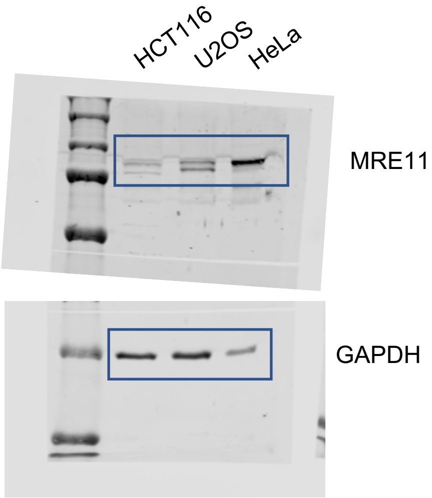

Supplement: Supplementary file 11 — Source Data EV Figures [file 44318_2024_38_MOESM11_ESM.zip › WBs FIG EVs.pdf]
