# Supplementary material for: RAD51 restricts DNA over-replication from re-activated origins
Source: EMBO J. 2024 Feb 15;43(6):7. doi: 10.1038/s44318-024-00038-z (PMC10942984; doi:10.1038/s44318-024-00038-z)
Supplement: Supplementary file 13 — Expanded View Figures [file 44318_2024_38_MOESM13_ESM.pdf]

Expanded View Figures

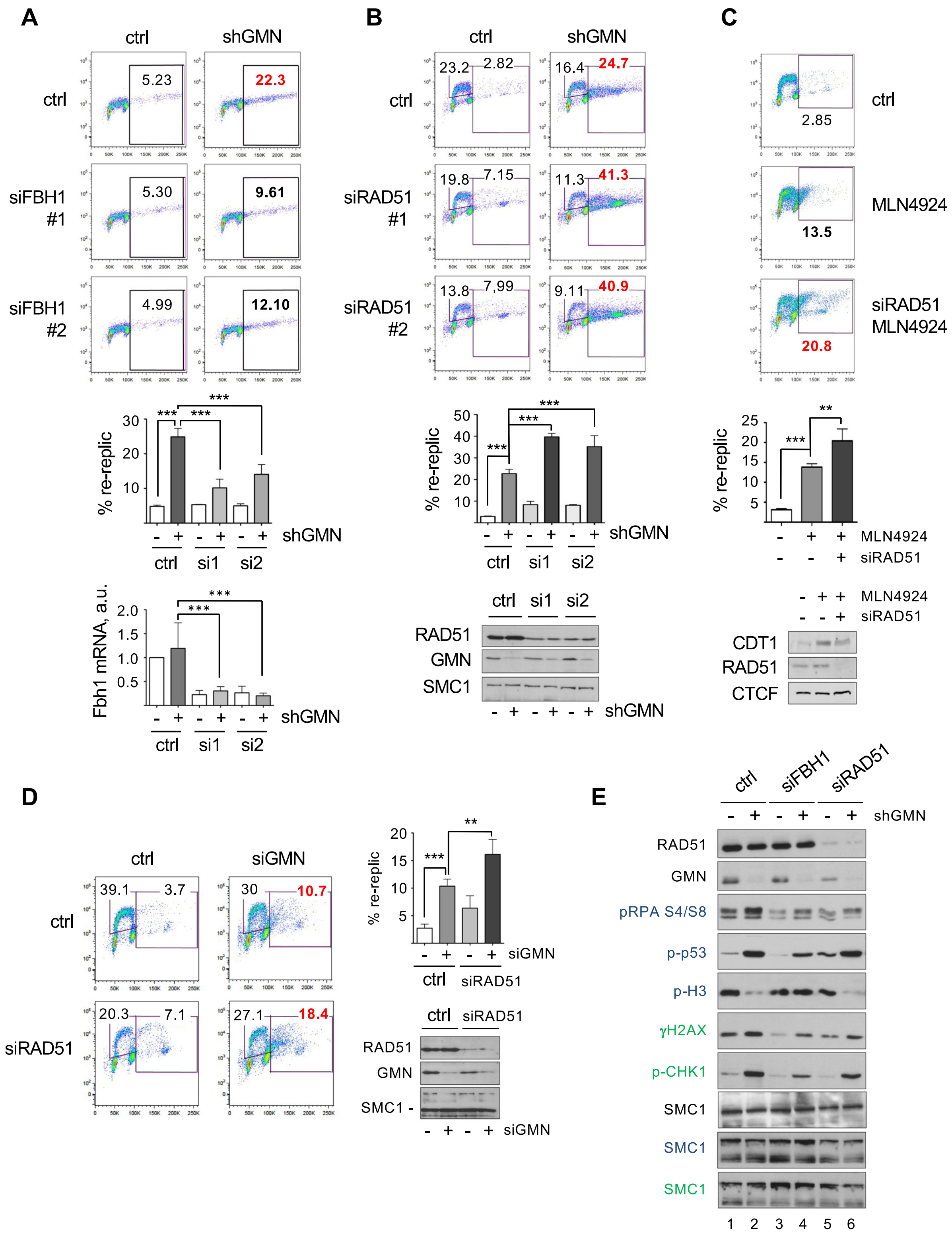

 © The Author(s)

Figure EV1. Opposing effects of RAD51 and FBH1 on DNA re-replication (related to Fig. 1).

(**A**) Top, analysis of re-replication in HCT116-shGMN cells treated with or without shGMN and transfected with the indicated FBH1 siRNAs for 72 h. Gates indicate cells with over-replicated DNA. Middle histogram shows the percentage (mean and SD) of cells undergoing re-replication. $n = 3$ replicates. ***$p < 0.001$ (one-way Anova and Bonferroni's post-test). Bottom histogram shows fold-change (mean and SD) of Fbh1 mRNA levels relative to control. $n = 3$ replicates. ***$p < 0.001$; one-way Anova and Bonferroni's post-test. (**B**) Same as (**A**), in HCT116-shGMN cells transfected with the indicated RAD51 siRNAs for 72 h. S-phase cells are included in the small gate, and cells with over-replicated DNA are included in the large gate. Middle histogram shows the percentage (mean and SD) of cells undergoing re-replication. $n = 3$ replicates. ***$p < 0.001$; one-way Anova and Bonferroni's post-test. Immunoblots show the levels of RAD51 and GMN proteins. SMC1 is shown as loading control. (**C**) Analysis of re-replication in HCT116-shGMN cells in the absence or presence of 250 nM MLN4924 and transfected with RAD51 siRNA for 72 h when indicated. Middle histogram shows the percentage (mean and SD) of cells undergoing re-replication. $n = 3$ replicates. ***$p < 0.001$; **$p < 0.01$ (one-way Anova and Bonferroni's post-test). Immunoblots show the levels of RAD51 and CDT1 proteins. CTCF is shown as loading control. (**D**) Analysis of re-replication in U2OS cells transfected with siGMN and/or siRAD51 for 72 h as indicated. Top right, histogram shows the percentage (mean and SD) of cells undergoing re-replication. $n = 3$ replicates. ***$p < 0.001$; **$p < 0.01$ (one-way Anova and Bonferroni's post-test). Bottom right, immunoblot detection of RAD51 and GMN proteins. SMC1 is included as loading control. (**E**) Immunoblot detection of the indicated proteins in HCT116-shGMN cells from the experiment shown in main Fig. 1D. SMC1 levels are shown as loading control. The three separate gels used to analyze this experiment are indicated by the legend color (black/green/blue). Source data are available online for this figure.

Figure EV2. HR factors and MUS81 do not regulate DNA re-replication (related to Fig. 2).

(**A**) Immunoblot detection of GMN in cells from the experiment described in main Fig. 2A, B. TUBULIN is shown as loading control. (**B**) Analysis of re-replication in HCT116-shGMN cells treated with or without shGMN for the indicated time. Histogram shows the percentage (mean and SD) of cells undergoing re-replication. $n = 3$ replicates. ***$p < 0.001$; n.s., not significant (one-way Anova and Bonferroni's post-test). The levels of GMN are shown by immunoblot. Tubulin is shown as loading control. (**C**) Percentage of cells displaying positive staining for γH2AX and chromatin-bound RAD51 in samples from main Fig. 2A. $n = 3$ replicates. **$p < 0.01$ (two-tailed, unpaired Student's t test). (**D**) Same as (**C**) for chromatin-bound RPA and RAD51 signals. $n = 3$ replicates. ***$p < 0.001$ (two-tailed, unpaired Student's t test). (**E**) Immunoblot detection of the RAD51AP1 and p-RPA32 (S4/S8) in HCT116-shGMN cells transfected with siRNA51AP1 and treated with 10 μM camptothecin (CPT) for 24 h as indicated. CTCF is shown as loading control. The two separate gels used to analyze this experiment are indicated by the legend color (black/blue). (**F**) Immunoblot levels of p21 protein in HCT116-shGMN cells treated with 10 μM B02 and/or 0.1 mM doxorubicin (DXR) for 24 h as indicated. SMC1 is shown as loading control. (**G**) Percentage of cells in each phase of the cell cycle, corresponding to the experiment shown in main Fig. 2E. $n = 3$ replicates. **$p < 0.01$ (two-tailed, unpaired Student's t test). Source data are available online for this figure.

© The Author(s)

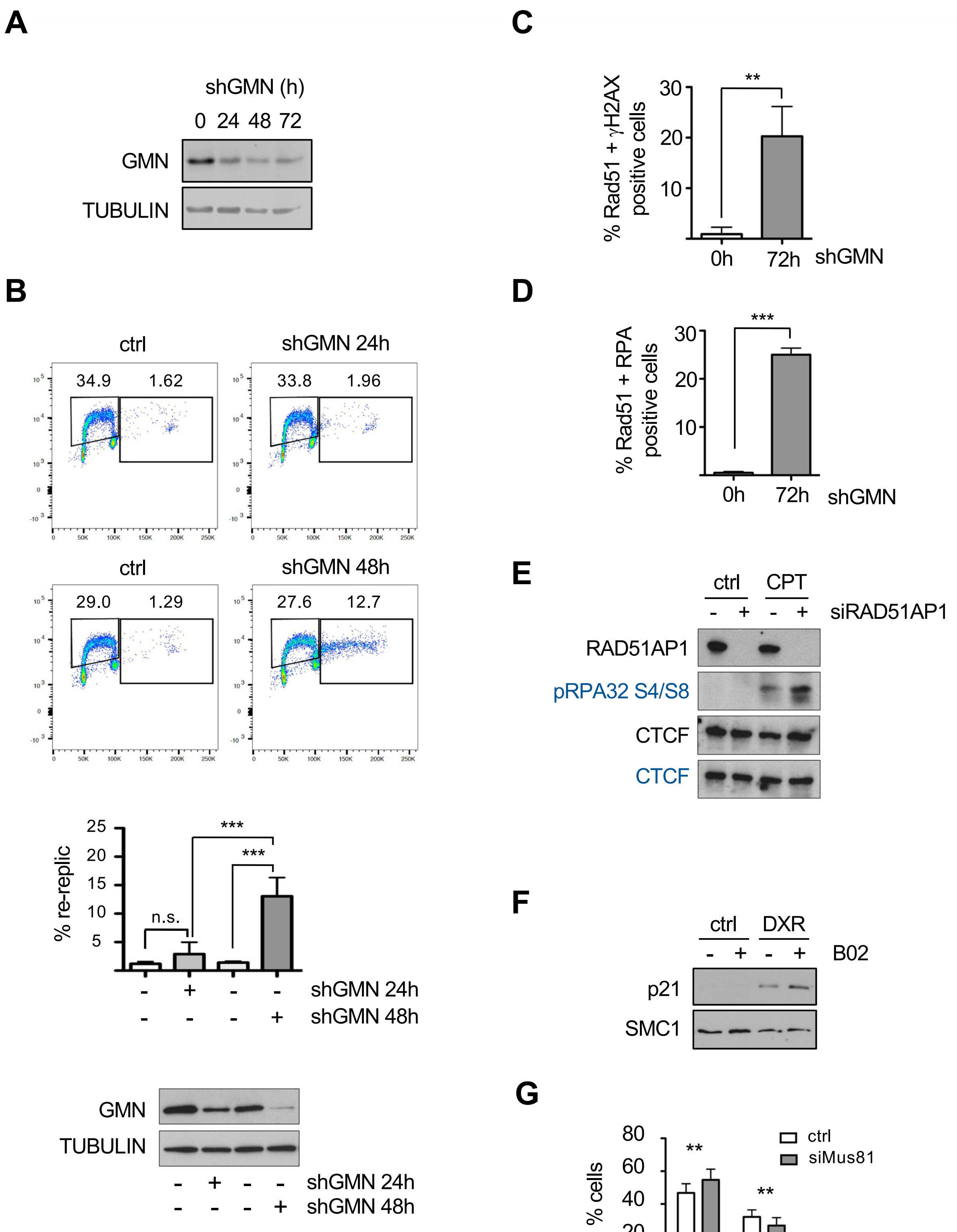
A
shGMN (h)
0 24 48 72
GMN
TUBULIN
B
ctrl
shGMN 24h
34.9 1.62
33.8 1.96
ctrl
shGMN 48h
29.0 1.29
27.6 12.7
% re-replic
n.s.
***
***
shGMN 24h
shGMN 48h
GMN
TUBULIN
shGMN 24h
shGMN 48h
C
% Rad51 + γH2AX positive cells
**
0h 72h shGMN
D
% Rad51 + RPA positive cells
***
0h 72h shGMN
E
ctrl CPT
siRAD51AP1
RAD51AP1
pRPA32 S4/S8
CTCF
CTCF
F
ctrl DXR
B02
p21
SMC1
G
% cells
ctrl
siMus81
**
**
G1 S G2/M

 © The Author(s)

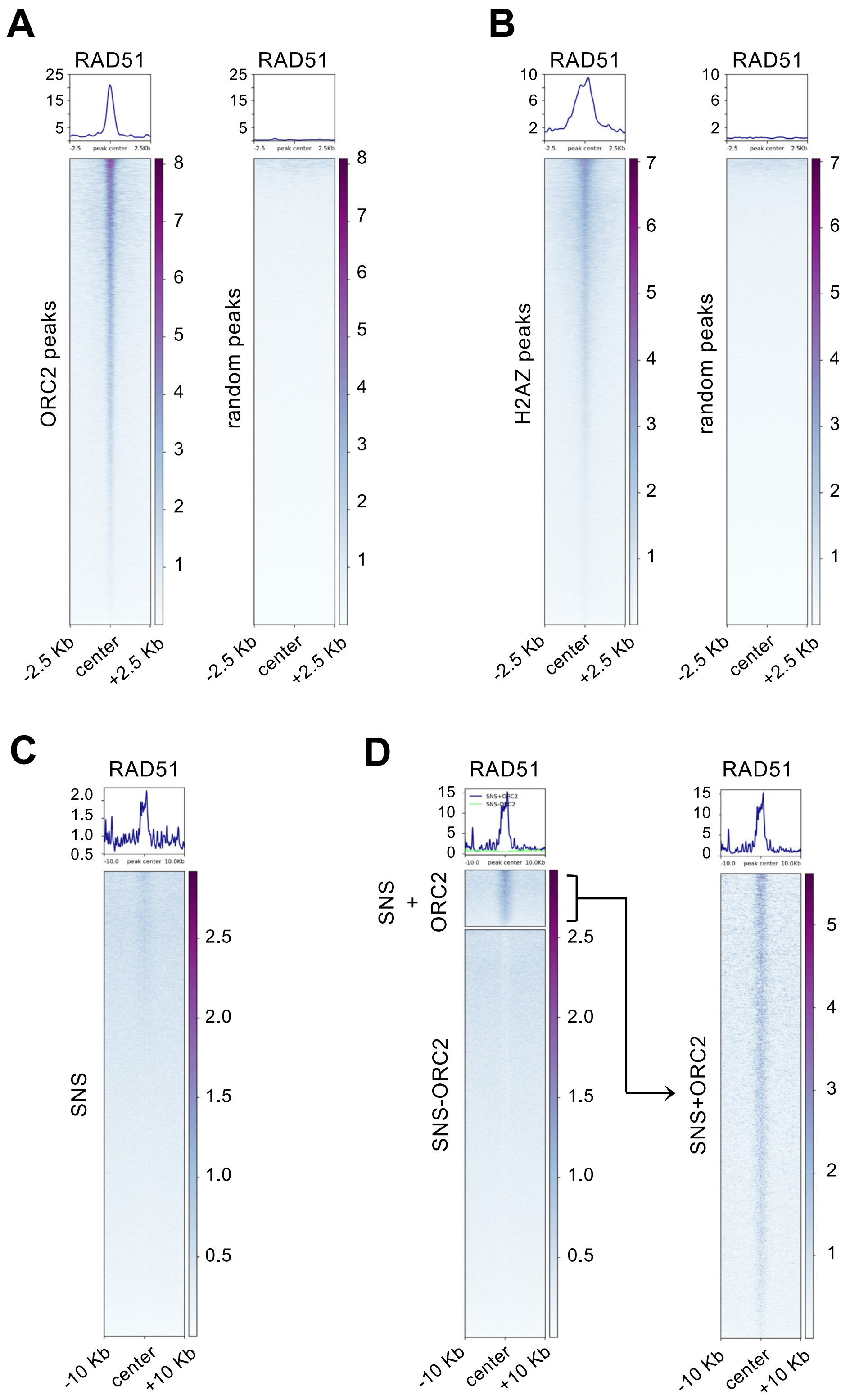

Figure EV3. RAD51 enrichment around replication origins (related to Fig. 3).

(**A**) Heatmap distribution of RAD51 ChIP-seq reads around ORC2-binding sites (left) or an equal number of randomized genomic positions (right). (**B**) Same as (**A**), showing the distribution of RAD51 around H2A.Z binding sites (left) or randomized genomic positions (right). (**C**) Heatmap distribution of RAD51 around all SNS-seq peaks. (**D**) Left, heatmap distribution of RAD51 around SNS-seq peaks stratified according to the simultaneous detection of ORC2 (SNS + ORC2 and SNS-ORC2 respectively). Right, blow-up of the distribution of RAD51 around SNS-seq peaks coincident with ORC2-binding sites.

© The Author(s)

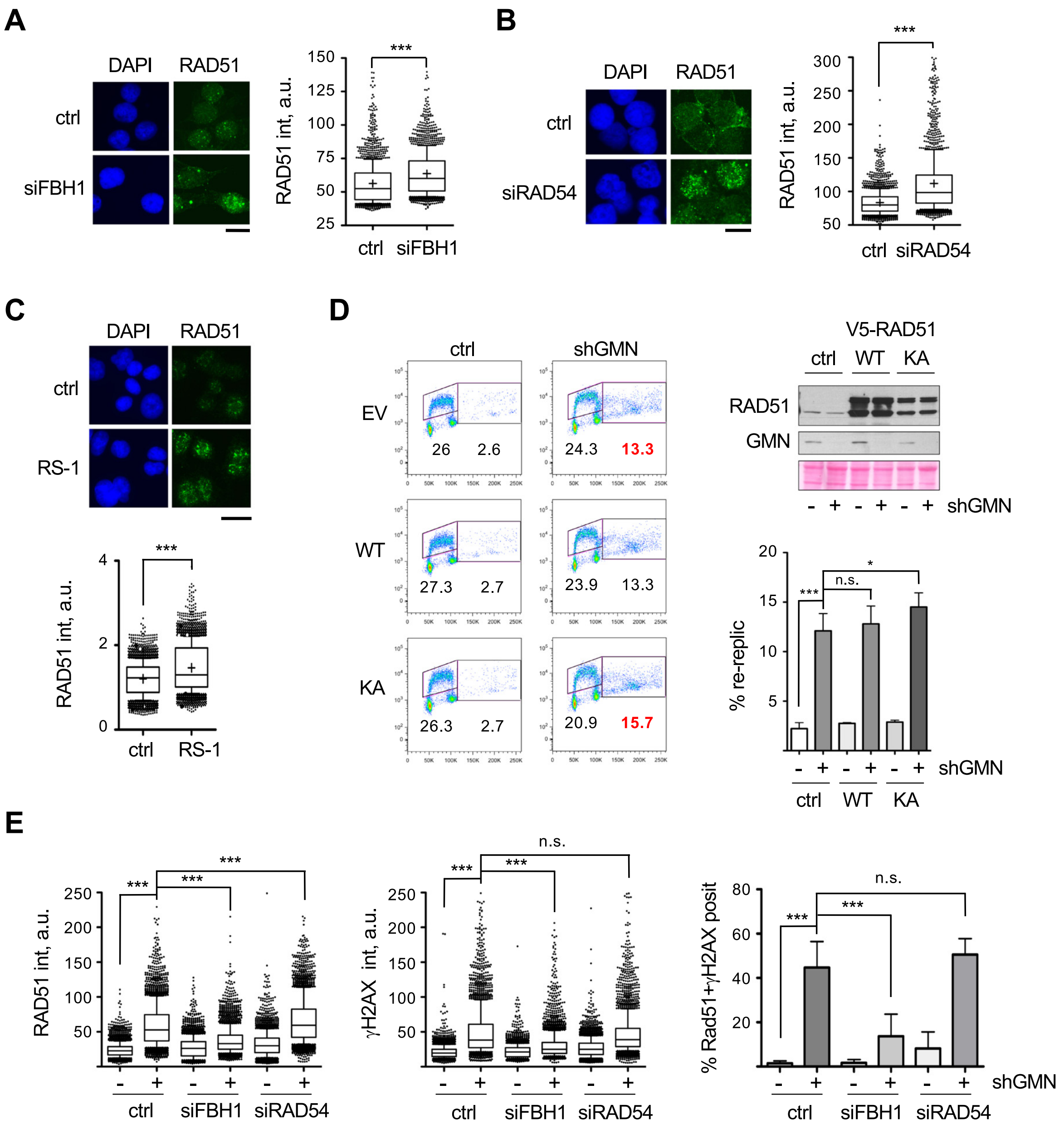

Figure EV4. Chromatin-bound RAD51 restricts DNA re-replication (related to Fig. 3).

(**A**) Representative IF images of HCT116-shGMN cells transfected when indicated with siFBH1 siRNA for 72 h, pre-extracted and immunostained with a different RAD51 antibody than the one used in main Fig. 3C. DNA was counterstained with DAPI (blue). Bar, 15 μm. Right, box and whiskers plot showing the distribution of chromatin-bound RAD51 intensity. Data from two experiments were pooled. $n = 1000$ cells per condition and replica. ***$p < 0.001$ (Mann–Whitney test). (**B**) Same as (**A**), in HCT116-shGMN cells transfected when indicated with RAD54 siRNA for 72 h. Bar, 15 μm. Data from two different experiments were pooled. $n = 1000$ cells per condition and replica. ***$p < 0.001$ (Mann–Whitney test). (**C**) Same as (**A**) and (**B**), in HCT116-shGMN cells treated with 7.5 μM RS1 for 24 h. Bar, 15 μm. Data from 4 different experiments were pooled. $n = 800$ cells per condition and replica. ***$p < 0.001$ (Mann–Whitney test). (**D**) Analysis of re-replication in HCT116-shGMN cells treated with or without shGMN and transfected with the indicated V5-tagged RAD51 variant for 72 h. Ctrl, empty vector; WT, V5-RAD51-WT; KA, V5-RAD51-K133A. Top right, immunoblot detection of RAD51 and GMN proteins. Red Ponceau-S staining of the membrane is shown as loading control. Bottom right, percentage (mean and SD) of cells undergoing re-replication. $n = 3$ replicates. ***$p < 0.001$; *$p < 0.05$; n.s., not significant (repeated measures Anova and Bonferroni's post-test). (**E**) Box and whiskers plots showing the distribution of RAD51 (left) and γH2AX (center) intensity in pre-extracted HCT116-shGMN cells treated with or without shGMN and transfected with the indicated siRNAs for 72 h. ctrl, siFBH1 and siRAD54 (-shGMN) samples are the same as in main Fig. 3C. Data from 3 different experiments are pooled. $n = 1000$ cells per condition and replica. ***$p < 0.0001$ (Kruskal–Wallis test and Dunn's post-test). Right histogram shows the percentage of cells displaying positive staining for γH2AX and chromatin-bound RAD51 in the indicated samples. $n = 3$. ***$p < 0.001$ (one-way Anova and Bonferroni's post-test). Box plots in (**A–C,E**): Boxes are drawn from the 25th to the 75th percentile. The central horizontal line indicates the median value. Whiskers are drawn from the 10th to the 90th percentile and the rest of values are drawn as individual dots (mimima, 0th percentile, maxima 100th percentile). In (**A–C**), the mean values are drawn as "+". Source data are available online for this figure.

 © The Author(s)

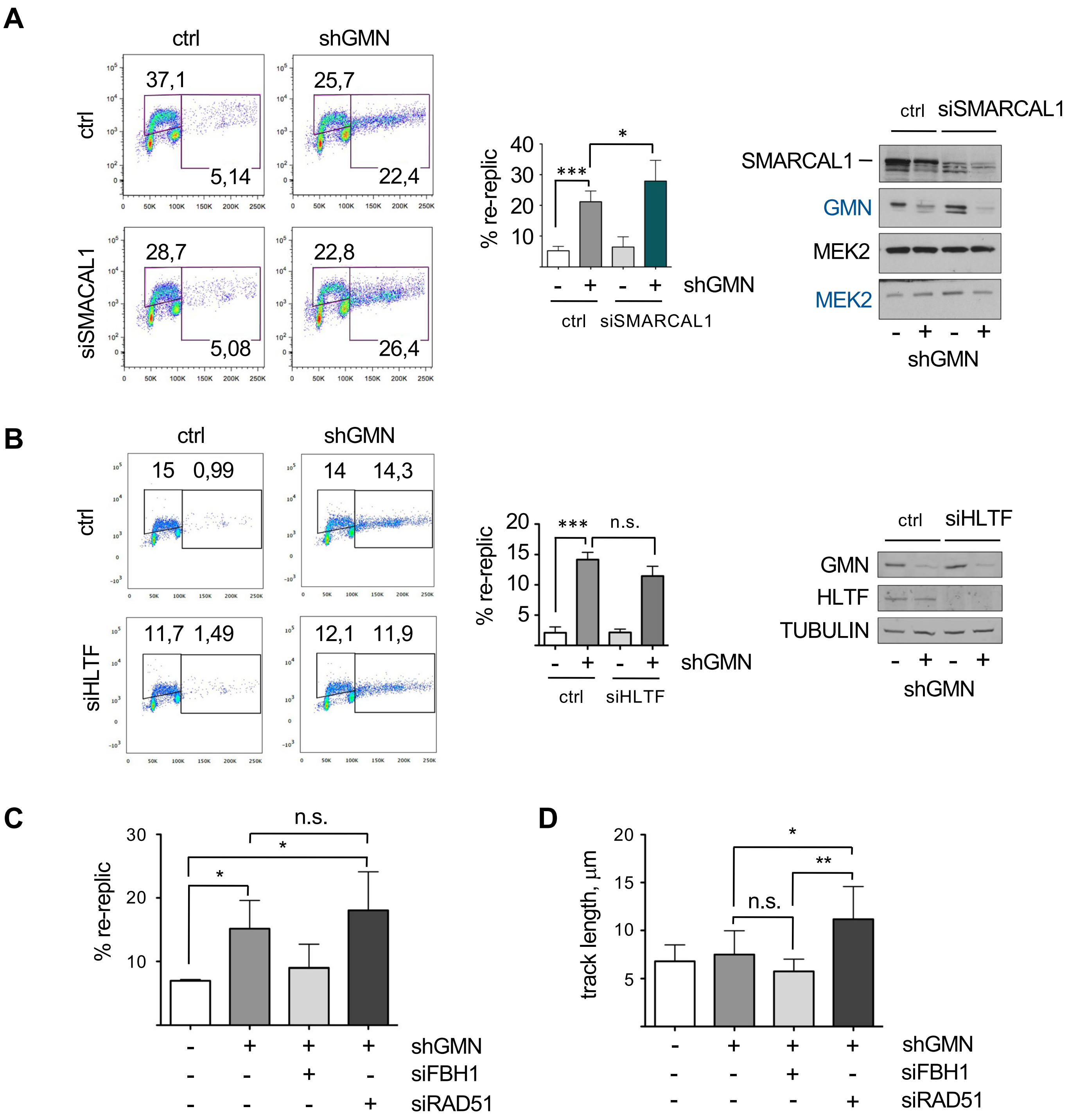

Figure EV5. RAD51 blocks the progression of re-replication forks (related to Fig. 4).

(**A**) Analysis of re-replication in HCT116-shGMN cells grown with or without shGMN and transfected when indicated with siSMARCAL1 for 72 h. Histogram shows the percentage (mean and SD) of cells undergoing re-replication. $n = 3$ replicates. ***$p < 0.001$; *$p < 0.05$ (repeated measures Anova and Bonferroni's post-test). Right, immunoblot detection of SMARCAL1 and GMN protein levels. MEK2 is shown as loading control. The two separate gels used to analyze this experiment are indicated by the legend color (black/blue). (**B**) Same as (**A**), in HCT116-shGMN cells transfected with siHLTF when indicated. $n = 3$ replicates. ***$p < 0.001$; n.s. not significant (repeated measures Anova and Bonferroni's post-test). Immunoblot shows HLTF and GMN protein levels, with TUBULIN as loading control. (**C**) Percentage (mean and SD) of re-replicated tracks relative to the total number of replicative structures from the experiment shown in main Fig. 4G. $n = 3$ replicates, with >394 replicative structures scored per condition and replicate. *$p < 0.05$; n.s., not significant (one-way Anova and Bonferroni's post-test). (**D**) Median length (mean and SD) of re-replicated tracks in samples from the experiment shown in main Fig. 4G. $n = 3$ replicates. **$p < 0.01$; *$p < 0.05$; n.s., not significant (one-way Anova and Bonferroni's post-test). Source data are available online for this figure.

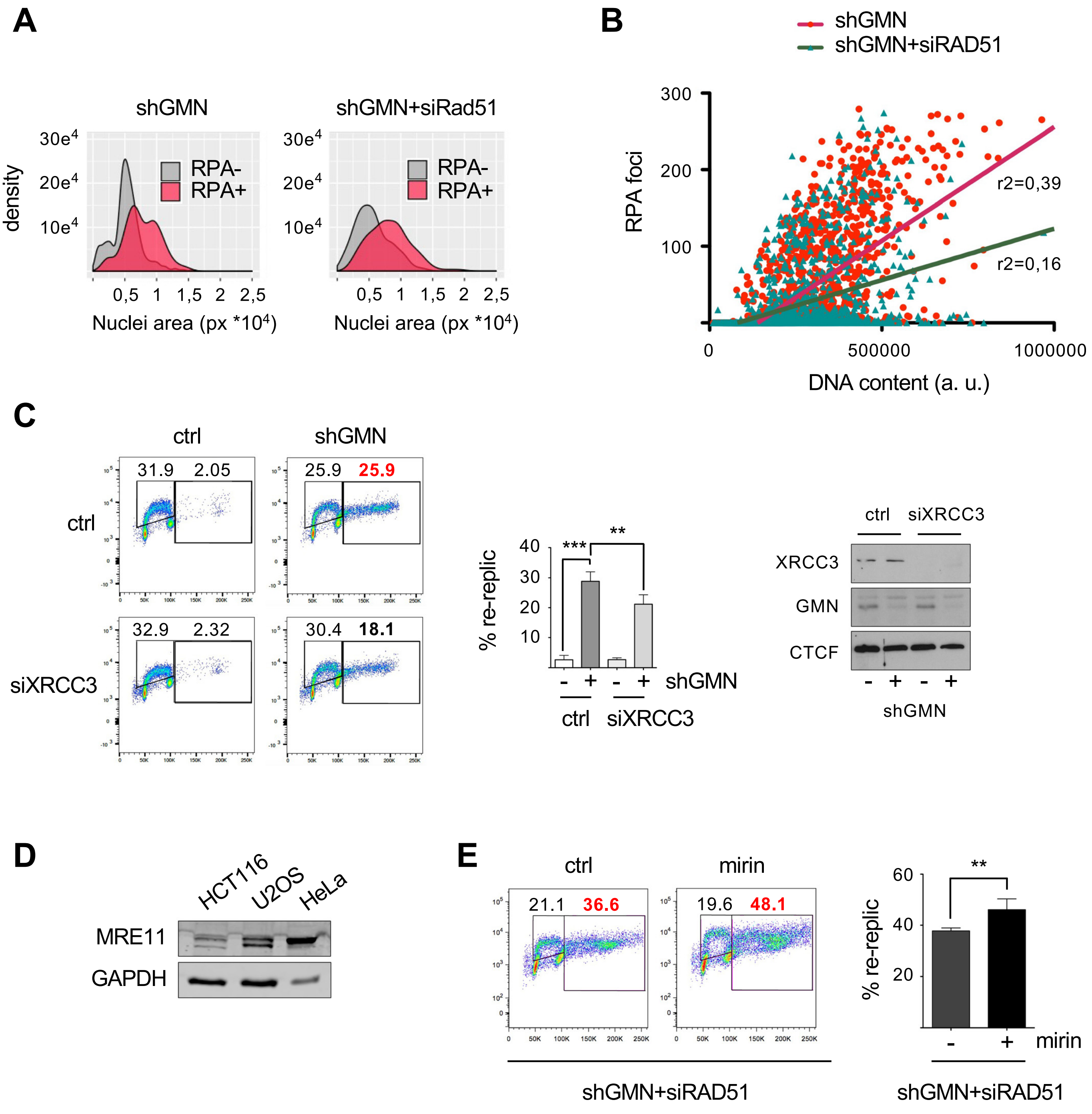

Figure EV6. Discontinuous DNA synthesis during re-replication (related to Fig. 5).

(**A**) Distribution of nuclear area in RPA-negative and RPA-positive cells (with or without siRAD51) derived from the experiment shown in main Fig. 5B. Cells with >10 RPA foci were considered positive. 450 cells from each condition were analyzed. One representative experiment (out of four) is shown. (**B**) Correlation between number of RPA foci and DNA content in the cells used in main Fig. 5B ($p < 0.001$ in Spearman non-parametric correlation test in both conditions). >1450 cells from each condition were analyzed. One representative experiment (out of four) is shown. The difference between slopes was statistically significant ($p < 0.001$ in linear regression test); R2 coefficients are indicated. (**C**) Analysis of re-replication in HCT116-shGMN cells grown with or without shGMN and transfected when indicated with XRCC3 siRNA for 72 h. Histogram shows the percentage (mean and SD) of cells undergoing re-replication. $n = 3$ replicates. ***$p < 0.001$; **$p < 0.01$ (one-way Anova and Bonferroni's post-test). Right, immunoblot detection of XRCC3 and GMN protein levels, with CTCF as loading control. (**D**) Immunoblot detection of MRE11 protein in different cell lines, with GAPDH as reference. (**E**) Analysis of re-replication in HCT116-shGMN cells transfected with siRAD51 (72 h). When indicated, 10 µM mirin was added for 24 h. Histogram shows the percentage (mean and SD) of cells undergoing re-replication. $n = 4$ assays. **$p < 0.01$ (Student's t-test). Source data are available online for this figure.

 © The Author(s)
